# Evaluation of Pulmonary Function in Long-Term Follow-Up After Laparoscopic Sleeve Gastrectomy

**DOI:** 10.1007/s11695-025-07672-4

**Published:** 2025-01-08

**Authors:** Hatice Şahin, Murat Şahin, Ertan Bülbüloğlu, Celal Kuş, Burcu Akkök, Nurhan Atilla

**Affiliations:** 1 Kahramanmaraş Sütçü İmam University, Kahramanmaraş, Turkey; 2 Bezmiâlem Vakıf Üniversitesi, Istanbul, Turkey

**Keywords:** Sleeve gastrectomy, Obesity, Pulmonary function, FEV1, PEF, MEF25-75

## Abstract

**Background:**

Obesity is one of the most important health problems in the world. It affects all systems, especially the respiratory and cardiovascular systems. Laparoscopic sleeve gastrectomy is an effective method in the treatment of obesity and can improve respiratory functions. We aimed to evaluate the effects of surgery on pulmonary function in patients with obesity.

**Methods:**

A retrospective analysis was conducted on a group of patients who underwent sleeve gastrectomy. This study assessed pre-operative and long-term pulmonary function in patients who underwent laparoscopic sleeve gastrectomy between 2009 and 2015, with a minimum follow-up of 10 years. Patients were stratified based on smoking status and presence of comorbidities.

**Results:**

The study included 51 patients (82.4% female) with a mean age of 51.90 ± 11.57 years. Significant weight loss and reductions in BMI were observed postoperatively. Mean pre-operative BMI was 47.53 ± 6.95 and significantly decreased to 37.75 ± 6.02 post-operatively, BMI reduction rate was %22 (*p* < 0.001). Pulmonary function tests demonstrated significant improvements in FEV1 (2.65 ± 0.69 to 2.76 ± 0.67, p = 0.044), FEV1% (92.07 ± 15.31 to 97.98 ± 14.45, *p* = 0.001), PEF (74.01 ± 18.12 to 91.53 ± 24.16, *p* < 0.001), and MEF25-75 (77.17 ± 22.07 to 108.57 ± 28.11 p < 0.001) after surgery. These improvements were consistent across different subgroups, including smokers, non-smokers, and patients with and without comorbidities. Non-smokers exhibited a greater percentage increase in FEV1 compared to smokers. While there was an increase in FEV1 among patients with comorbidities, this difference was not statistically significant. Conversely, patients without comorbidities demonstrated a significant improvement in FEV1.

**Conclusion:**

Bariatric surgery is associated with significant improvements in pulmonary function in obese patients, regardless of smoking status or comorbidities.

## 1-Introduction

Obesity is a global public health problem today and can lead to many diseases such as diabetes, heart disease, and cancer [1]. Obesity surgery is the most effective treatment method for weight loss in patients with morbid obesity.

In 2022, 2.5 billion adults (18 years and older) were overweight, of which 890 million were living with obesity, representing 43% and 16% of the adult population, respectively [2].

Obesity prevalence was found to be 41% in women, 20.5% in men, and 30.3% in the overall population [3]. As a chronic disease with increasing global prevalence, obesity is expected to increase to 51% worldwide by 2030 [4].

Obesity has far-reaching systemic effects. Long-term consequences include increased risks of cardiovascular disease, diabetes mellitus, and various cancers. The respiratory system is significantly impacted by obesity. Obesity is a well-established risk factor for obstructive sleep apnea, leading to increased fat deposition in the pharyngeal airways [5]. Moreover, obese individuals exhibit a higher prevalence, severity, and hospitalization rate for asthma [6]. Obesity hypoventilation syndrome, a respiratory disorder characterized by inadequate ventilation, is another common complication in obese individuals, affecting 8–20% of this population [7].

While dietary and pharmacological interventions are commonly employed in obesity management, suboptimal outcomes are frequent. Bariatric surgery has emerged as the gold standard for long-term weight management in severely obese individuals, inducing significant and sustained weight loss [8]. Among bariatric surgical procedures, laparoscopic sleeve gastrectomy is widely accepted as a frequently performed and highly effective method. After sleeve gastrectomy, patients often experience remission of obesity-related comorbidities such as type 2 diabetes, hypertension, and cardiovascular disease. Additionally, improvements in respiratory conditions, including obstructive sleep apnea, are commonly reported [9].

Although there have been numerous studies investigating the effects of bariatric surgery on respiration in the early period, long-term follow-up studies are very limited. The aim of our study is to investigate the effect of bariatric surgery on respiratory function 10 years after surgery in patients who were evaluated preoperatively in our clinic for bariatric surgery.

## 2-Material and Method

This cross-sectional study aimed to assess the pre-operative and long-term (at least 10 years) pulmonary function in patients who underwent laparoscopic bariatric surgery at Kahramanmaraş Sütçü İmam University between 2009 and 2015. A total of 183 patients aged 18 or older who had undergone bariatric sleeve gastrectomy between 2009 and 2015 were approached for our study. Following phone calls, 75 patients declined to participate, 5 had passed away, and 52 could not be reached. Consequently, 51 patients who consented to participate were enrolled in the study. Ethical approval for this study was obtained from the Non-Interventional Clinical Research Ethics Committee of Kahramanmaraş Sütçü İmam University on March 14, 2024 (approval number 58).

The long-term impact of bariatric surgery on respiratory function tests (RFT) was investigated in participants who fulfilled the inclusion criteria, agreed to the study conditions, and provided informed consent. Specifically, the study examined the effects of surgery on respiratory function parameters such as FEV1, FVC, FEV1/FVC ratio, peak expiratory flow (PEF), and mean flow between 25 and 75% of forced vital capacity (MEF25-75). These parameters were extracted from the participants’ medical records, which also included data on body mass index, age, comorbidities, and smoking history.

### 1-Statistical Analysis

Data were analyzed using SPSS version 20.0. The normality of the data was assessed using visual methods (histograms and probability plots) and analytical tests (Kolmogorov–Smirnov and Shapiro–Wilk tests). Descriptive statistics were presented as mean ± standard deviation for continuous variables with normal distribution and as median, minimum, and maximum for those without. For categorical variables, frequency (*n*) and percentage (%) were reported.

For continuous data collected from repeated measurements of the same subjects, paired *t*-tests were employed when data followed a normal distribution. For non-normal data, the Wilcoxon signed-rank test was used. The Spearman correlation test was conducted to assess the relationship between variables. The correlation table below summarizes the relationship status. Statistical significance was set at *p* ≤ 0.05.

According to the power analysis, the effect size was found to be 0.454 based on the values of obese patients who underwent obesity surgery in the reference study [10]. Considering an alpha of 0.05 for a type I error, a beta of 0.20 for a type II error, a power of 0.80, and an effect size of 0.454, the calculation indicated that n:41 patients were planned to be included in the study.

## 3-Findings

The descriptive and sociodemographic characteristics of the participants are presented in Table 1. The mean age of the patients was 51.90 ± 11.57 years (median 56.0, range 27.0–72.0) (*p* < 0.001). Nine patients (17.6%) were male, and 42 (82.4%) were female. Upon further examination, it was found that 13 (26.5%) of the participants were smokers, and 23 (45.1%) had comorbidities.

**Table 1:** Sociodemographic and spirometry characteristics

Characteristic	Pre-surgery	Post-surgery	*p*
Age	41.43 ± 11.31	51.90 ± 11.57	< 0.001**
Weight	126.62 ± 16.97	100.78 ± 16.56	< 0.001*
BMI	47.53 ± 6.95	37.75 ± 6.02	< 0.001*
FEV1 (L)	2.65 ± 0.69	2.76 ± 0.67	0.044**
FEV1 (%)	92.07 ± 15.31	97.98 ± 14.45	0.001*
FVC (L)	3.31 ± 0.80	3.40 ± 0.77	0.116*
FEV1/FVC	80.94 ± 7.40	81.22 ± 6.37	0.781*
PEF	74.01 ± 18.12	91.53 ± 24.16	< 0.001**
MEF25-75	77.17 ± 22.07	108.57 ± 28.11	< 0.001**

^*^Paired *T*-test, **Wilcoxon test

*BMI* body mass index**, ***FEV1* forced expired volume in 1 s, *FVC* forced vital capacity, *PEF* peak expiratory flow, *MEF25-75* maximum expiratory flow 25–75% of FVC

Patients had a mean pre-operative weight of 126.62 ± 16.97 kg, which significantly decreased to 100.78 ± 16.56 kg following surgery (*p* < 0.001) (Table 1). Likewise, mean pre-operative BMI was 47.53 ± 6.95 and significantly decreased to 37.75 ± 6.02 post-operatively; BMI reduction rate was %22 (*p* < 0.001) (Table 1).

The FEV1 (in liters) significantly increased from 2.65 ± 0.69 before surgery to 2.76 ± 0.67 after surgery (*p* = 0.044). The FEV1 (% predicted) value also significantly increased from 92.07 ± 15.31 before surgery to 97.98 ± 14.45 after surgery (*p* = 0.001) (Table 1).

In smokers, FEV1 (L) increased from 2.65 ± 0.69 to 2.76 ± 0.67 (*p* = 0.124), and FEV1 (%) also increased significantly from 95.07 ± 11.84 to 99.69 ± 13.29 (*p* < 0.016). After surgery, FVC (L) in smoking patients increased from 3.25 ± 0.38 to 3.37 ± 0.46 (*p* = 0.178), but there was no significant change in FEV1/FVC (*p* = 0.717). The PEF value in smokers increased from 77.92 ± 18.31 before surgery to 88.85 ± 18.64 after surgery (*p* < 0.069). Similarly, the MEF25-75 value, which was 73.61 ± 15.06 before surgery, increased significantly to 102.54 ± 23.91 (*p* = 0.001) (Table 2).

**Table 2:** RFT characteristics according to surgery status in smokers

	**Smokers pre-surgery**	**Smokers post-surgery**	***p***
FEV1 (L)	2.61 ± 0.38	2.69 ± 0.39	0.124**
FEV1 (%)	95.07 ± 11.84	99.69 ± 13.29	0.016*
FVC (L)	3.25 ± 0.38	3.37 ± 0.46	0.178*
FEV1/FVC	80.30 ± 5.29	79.85 ± 4.58	0.717*
PEF	77.92 ± 18.31	88.85 ± 18.64	< 0.069**
MEF25-75	73.61 ± 15.06	102.54 ± 23.91	0.001**

^*^Paired *T*-test, **Wilcoxon test

*FEV1* forced expired volume in 1 s, *FVC* forced vital capacity, *PEF* peak expiratory flow, *MEF25-75* maximum expiratory flow 25–75% of FVC

In non-smokers, FEV1(L) increased from 2.67 ± 0.78 to 2.78 ± 0.74 (*p* = 0.105), and FEV1 (%) significantly increased from 91.05 ± 16.35 pre-operatively to 97.39 ± 14.95 post-operatively (*p* = 0.005). Pre-operative FVC(L) value of 3.33 ± 0.91 increased to 3.40 ± 0.85 (*p* = 0.265). There was no significant change in FEV1/FVC (*p* = 0.683). PEF significantly increased from 72.69 ± 18.11 to 92.44 ± 25.94 (*p* < 0.001). MEF25-75 also significantly increased from 78.39 ± 24.06 to 110.64 ± 29.41 (*p* < 0.001) (Table 3).

**Table 3:** RFT characteristics according to surgery status in non-smokers

Feature	Non-smokers pre-surgery	Non-smokers post-surgery	*p*
FEV1 (L)	2.67 ± 0.78	2.78 ± 0.74	0.105**
FEV1 (%)	91.05 ± 16.35	97.39 ± 14.95	0.005*
FVC (L)	3.33 ± 0.91	3.40 ± 0.85	0.265*
FEV1/FVC	81.16 ± 8.05	81.70 ± 6.86	0.683*
PEF	72.69 ± 18.11	92.44 ± 25.94	< 0.001**
MEF25-75	78.39 ± 24.06	110.64 ± 29.41	< 0.001**

^*^Paired *T*-test, **Wilcoxon test

*FEV1* forced expired volume in 1 s, *FVC* forced vital capacity, *PEF* peak expiratory flow, *MEF25-75* maximum expiratory flow 25–75% of FVC

In patients with concomitant diseases, pre-operative FEV1 (L) increased from 2.39 ± 0.63 to 2.44 ± 0.49 (*p* = 0.784), and FEV1 (%) increased from 88.78 ± 15.04 to 92.95 ± 12.86 (*p* = 0.184). Pre-operative FVC (L) increased from 3.02 ± 0.66 to 3.03 ± 0.55 (*p* = 0.973). There was no significant change in FEV1/FVC (*p* = 0.906). Pre-operative PEF, which was 69.34 ± 19.94, significantly increased to 93.86 ± 31.45 (*p* < 0.001). Pre-operative MEF25-75, which was 72.91 ± 18.67, significantly increased to 102.86 ± 26.56 (*p* < 0.001) (Table 4).

**Table 4:** RFT characteristics according to surgical status in patients with comorbidities

	**Pre-surgery in patients with comorbidities**	**Post-surgery in patients with comorbidities**	***p***
FEV1 (L)	2.39 ± 0.63	2.44 ± 0.49	0.784**
FEV1 (%)	88.78 ± 15.04	92.95 ± 12.86	0.184*
FVC (L)	3.02 ± 0.66	3.03 ± 0.55	0.973*
FEV1/FVC	80.52 ± 6.91	80.28 ± 7.71	0.906*
PEF	69.34 ± 19.94	93.86 ± 31.45	0.001**
MEF25-75	72.91 ± 18.67	102.86 ± 26.56	< 0.001**

^*^Paired *T*-test, **Wilcoxon test

*FEV1* forced expired volume in 1 s, *FVC* forced vital capacity, *PEF* peak expiratory flow, *MEF25-75* maximum expiratory flow 25–75% of FVC

In patients without concomitant diseases, FEV1 (L) increased significantly from 2.87 ± 0.69 to 3.03 ± 0.68 before and after surgery, respectively (*p* = 0.002). FEV1 (%) increased significantly from 94.78 ± 15.28 to 102.11 ± 14.59 (*p* < 0.001). FVC (L) increased from 3.54 ± 0.84 to 3.70 ± 0.81 (*p* < 0.025). There was no significant change in FEV1/FVC (*p* = 0.466). PEF increased significantly from 77.85 ± 15.81 to 89.61 ± 16.35 after surgery (*p* = 0.001). MEF25-75 increased significantly from 80.67 ± 24.29 to 113.27 ± 28.95 after surgery (*p* < 0.001) (Table 5).

**Table 5:** RFT characteristics according to surgery status in patients without comorbidity

Feature	Pre-surgery in patients without comorbidity	Post-surgery in patients without comorbidity	*p*
FEV1 (L)	2.87 ± 0.69	3.03 ± 0.68	0.002**
FEV1 (%)	94.78 ± 15.28	102.11 ± 14.59	< 0.001*
FVC (L)	3.54 ± 0.84	3.70 ± 0.81	0.025*
FEV1/FVC	81.29 ± 7.89	82.01 ± 5.05	0.466*
PEF	77.85 ± 15.81	89.61 ± 16.35	0.001**
MEF25-75	80.67 ± 24.29	113.27 ± 28.95	< 0.001**

^*^Paired *T*-test, **Wilcoxon test

*FEV1* forced expired volume in 1 s, *FVC* forced vital capacity, *PEF* peak expiratory flow, *MEF25-75* maximum expiratory flow 25–75% of FVC

## 4-Discussion

Obesity is a common problem and prevalence is higher in women. Of the patients in our study, 17.6% were male and 82.4% were female. A 2015 study reported a higher prevalence of obesity among women compared to men. The predominance of women among patients undergoing laparoscopic gastric bypass surgery for obesity aligns with the higher prevalence of morbid obesity in women [11].

Bariatric surgery is the most effective and sustainable treatment for weight loss in obesity.

A review of 10 + years of laparoscopic sleeve gastrectomy results found that patients typically lose an average of 24.4% of their total body weight [12]. Similarly, our patients lose weight from 126.62 ± 16.97 kg to 100.78 ± 16.56 kg.

Patients with morbid obesity experience both restrictive and obstructive breathing impairments. Fat deposition in the thoracic and abdominal cavities reduces lung compliance, resulting in restrictive lung disease [13]. Concurrently, upper airway obstruction caused by adipose tissue accumulation contributes to obstructive lung disease. The increased soft tissue mass associated with obesity exerts pressure on the chest wall and elevates pulmonary blood flow. Both forced expiratory volume in one second (FEV1) and forced vital capacity (FVC) are diminished in obese individuals, although the FEV1/FVC ratio typically remains unaffected. Obese patients exhibit tachypnea and reduced tidal volumes. Airway resistance is elevated in this population [14].

Our research has shown that sleeve gastrectomy significantly improves all RFT parameters. Obesity induces various physiological alterations that compromise lung capacity and function. By facilitating weight loss and reversing these adverse changes, obesity surgery enhances pulmonary function.

MEF25-75 is employed to diagnose small airway obstruction. PEF offers insights into large airway obstruction [15]. The quantity of adipose tissue within the airways is correlated with BMI in humans. This indicates to us that the presence of adipose tissue within the airway wall modifies airway conduct [16]. Research has demonstrated a higher prevalence of asthma in obese individuals compared to non-obese individuals [17].

Weight gain causes an increase in airway resistance due to the mechanical effect of obesity on the respiratory system. Numerous studies conducted in Europe and America have shown that airway resistance is higher in obese adults compared to lean adults [14]. Beyond its mechanical impacts, obesity-induced inflammation is recognized as a contributing factor to respiratory diseases. Adipose tissue is an active endocrine organ that secretes various hormones and cytokines and is closely related to inflammation. Many studies have shown that obesity is a risk factor for chronic respiratory diseases, especially asthma and chronic obstructive pulmonary disease [18]. Weight reduction in obese individuals can reverse these findings, thereby improving respiratory findings in patients. In one study, 71 patients were evaluated 1 year after laparoscopic sleeve gastrectomy, and significant increases were observed in lung volumes and respiratory symptoms [19]. In another study of 68 patients who underwent bariatric sleeve gastrectomy surgery and were compared again 1 year later in terms of respiratory function, significant increases were observed in FEV1, FVC, PEF, and MEF25-75 values [20].

A key finding of our study is that laparoscopic sleeve gastrectomy surgery led to a significant improvement in patients’ pulmonary function, as evidenced by marked increases in FEV1, FEV1%, PEF, and MEF25-75 (*p* ≤ 0.05) (Table 1). These results suggest that laparoscopic sleeve gastrectomy surgery can substantially enhance airflow and lung capacity in individuals with obesity. While FEV1/FVC showed a non-significant trend towards improvement, FVC and FVC% increased without reaching statistical significance (*p* > 0.05) (Table 2). The significant long-term increase in FEV1 following weight loss may be attributed to the attenuation of chronic inflammatory processes associated with obesity.

Cigarette smoking induces widespread alterations in lung tissue and reduces respiratory volume [21]. A noteworthy finding of our study is that sleeve gastrectomy surgery yields superior improvements in all RFT parameters among non-smokers relative to smokers. This indicates that laparoscopic bariatric surgery has the potential to enhance respiratory function, even in individuals who smoke.

This study has several limitations. First, its retrospective design and relatively small sample size limit the generalizability of the findings. Second, the lack of long-term follow-up data, including weight loss maintenance and serial pulmonary function tests, prevents a comprehensive assessment of the long-term impact of bariatric surgery on pulmonary function. Future research should investigate the potential for further research to investigate hormonal changes associated with different bariatric procedures, as these may contribute to variations in postoperative pulmonary function outcomes. Additionally, prospective studies with larger sample sizes and longer follow-up periods are needed to confirm these findings and explore the potential mechanisms underlying the observed improvements in pulmonary function after bariatric surgery.

## 5-Conclusion

Sleeve gastrectomy is an effective method for treating morbid obesity and also reduces the risk of chest diseases in the long term. Sleeve gastrectomy has a positive impact on respiratory function. Therefore, sleeve gastrectomy can be considered as a treatment option to reduce the risk of chest diseases in patients with morbid obesity.

## Data Availability

No datasets were generated or analysed during the current study.
